# Early versus delayed silo closure in gastroschisis: a retrospective study

**DOI:** 10.1007/s00383-025-06042-6

**Published:** 2025-05-18

**Authors:** Pragati Sharma, Parshotam Gera, Shripada Rao

**Affiliations:** 1 Department of Paediatric Surgery, Perth Children’s Hospital, Nedlands, WA Australia; 2 Department of Neonatology, Perth Children’s Hospital, Nedlands, WA Australia; 3 School of Medicine, University of Western Australia, Crawley, WA Australia

**Keywords:** Gastroschisis, Simple, Complex, Neonatal, Silo, Outcomes, Early, Late

## Abstract

**Background:**

A well accepted approach to the management of gastroschisis is gradual reduction of the herniated viscera using preformed silos followed by surgical closure of the abdominal wall defect. However, if the abdominal wall closure is delayed for a longer duration than necessary, it may increase morbidities. We sought to compare the outcomes of infants undergoing silo reduction whose abdominal wall defect was closed ≤ 5 days versus > 5 days after birth.

**Methods:**

Retrospective cohort study (January-2010 to December-2020).

**Results:**

One-hundred-and-nine infants who were managed using primary silo with staged reduction were included. Median gestation was 36.2 (interquartile range, IQR: 35.2, 37) weeks. Ten infants had complex gastroschisis. Thirty-four infants underwent early-closure of abdominal wall defect and 75 had delayed closure. Mortality rate was 2.7% (3/109; one in early and two in delayed closure). The median age at full feeds was 24.5 days (IQR 17.5, 30) in the delayed-closure group vs 15 (12.5, 22.5) in the early-closure group. The median hospital stay was 32 days (IQR 23, 43) vs 19 (15, 30) days. On multivariable analysis, delayed closure (Exponentiated regression coefficient ERC 1.40, 95% confidence interval CI: 1.05, 1.86, *P* = 0.020) and complex gastroschisis (ERC 2.03; 95% CI: 1.11, 3.72, *P* = 0.021) were associated with longer time to reach full feeds. Same factors were associated with longer duration of hospital stay.

**Conclusions:**

Gradual reduction using silos achieved excellent outcomes in neonates with gastroschisis. Completing the silo reduction and closing the abdominal wall within five days could further improve their outcomes.

## Background

Gastroschisis is defined as a defect in the abdominal wall to the right of the umbilicus with herniated abdominal contents lacking an overlying covering [1]. The recent report from the International Clearinghouse for Birth Defects Surveillance and Research (ICBDSR) found a prevalence of 3.06 per 10,000 births; 95% confidence intervals [CI]: 3.01, 3.11), with marked regional variation. European prevalence was 1.49 (95%CI: 1.44, 1.55), Latin American 3.80 (95%CI: 3.69, 3.92) and North American 4.32 (95%CI: 4.22, 4.42). It also observed a statistically increased prevalence over time [2].

Gastroschisis can be classified as simple or complex, based on the presence or absence of additional complications such as atresia, perforation, ischaemia, necrosis, or volvulus noted at or immediately after birth. Complex gastroschisis is associated with increased time to feeds and length of stay and other morbidities [3–5]. Polyhydramnios on third trimester prenatal ultrasound on babies with gastroschisis can predict complex gastroschisis at birth [4].

The postnatal surgical management of gastroschisis involves reducing the herniated viscera back into the abdomen and closing the abdominal wall defect. The two main approaches to achieve this are a) primary reduction of the herniated viscera into the abdomen immediately after birth and surgical closure of the defect or b) gradual reduction of the herniated viscera using preformed silos followed by surgical closure of the abdominal wall after few days. In either of the methods, if the defect is large, a mesh patch is used to close the abdominal fascial wall.

Two small RCTs (total sample size: 92) that compared primary reduction versus staged reduction using preformed silo found no difference in clinical outcomes [6, 7]; however, one of them found a trend towards decreased ventilator days in the silo group [6]. Our experience in the past was that primary closure without using silo was associated with worse outcomes [8]. Since 2010, the standard practice of our unit is gradual reduction of herniated bowel using preformed silos followed by surgical closure of the abdominal wall defect. Whilst this approach is safe and effective, if the surgical closure of abdominal wall is delayed for longer than necessary, it may result in increased morbidities and prolong the hospital stay. A recent retrospective study found that closing the silo within five days was associated with better outcomes [9].

### Aims and objectives

1. To describe the clinical outcomes of infants with gastroschisis managed with preformed silos and
2. To compare the outcomes of gastroschisis infants whose abdominal wall defect was closed within ≤ 5 days versus > 5 days.

## Methods

It was a retrospective study of all infants with gastroschisis admitted between January 2010 and December 2020 to the neonatal intensive care unit of Perth Children’s Hospital in Western Australia and managed with silos. The conduct of this study was approved by our Institutional Ethics Committee as a clinical audit [Reference number 35119]. Informed parental consent was deemed not necessary since it was a retrospective study based on chart review.

Cases were identified by interrogating the surgical and neonatal databases of the hospital. Relevant clinical details during hospital stay were obtained by reviewing the medical charts of the cases. The infants were classified into two groups based on the postnatal day when the abdominal wall defect was closed (≤ 5 days vs > 5 days).

The following clinical information was collected from all study infants until discharge from hospital: gestation, birth weight, sex (male/female), mode of delivery, place of delivery, birth centiles, type of gastroschisis (simple/complex), size of silo, use of mesh for abdominal wall closure, duration of ventilation, antibiotic duration, time of commencing feeds, time to reach full feeds, duration of parenteral nutrition, duration of hospital stay, discharge weight and centiles, sepsis, wound infections, gut necrosis, abdominal compartment syndrome, and mortality during initial hospitalisation.

### Statistical analysis

Statistical analysis was conducted using Stata 18.0 (StataCorp. 2024. Stata Statistical Software: Release 18. College Station, TX: StataCorp LLC). Mean and standard deviation were used to summarise normally distributed data. Median and IQR were used to summarise data with skewed distribution. We conducted univariable and multivariable regression analyses using Generalised Linear Model with Gamma Distribution and log link function to evaluate the association between clinical factors and time to achieve full enteral feeds and duration of hospital stay. This model was chosen since the outcomes of interest (i.e. time to reach full enteral feeds and duration of hospital stay) were non-negative, continuous, and positively skewed data. The following potential confounders were adjusted for in the multivariable analysis: 1. gestational age, 2. sex, 3. birth weight, 4. birth weight z-score, 5. Type (complex or simple gastroschisis), 6. use of mesh patch to close the abdominal wall defect, 7. pre-operative ventilation, 8. time taken to commence enteral feeds after closing the abdominal wall and 9. bowel in silo for > 5 days vs ≤ 5 days prior to abdominal wall closure. The exponentiated coefficients from the regression analysis along with their respective 95% confidence intervals are reported. For all results, a two tailed p-value of < 0.05 was considered statistically significant. The results of the study are reported in accordance with the STROBE guidelines [10].

## Results

**Fig. 1:**
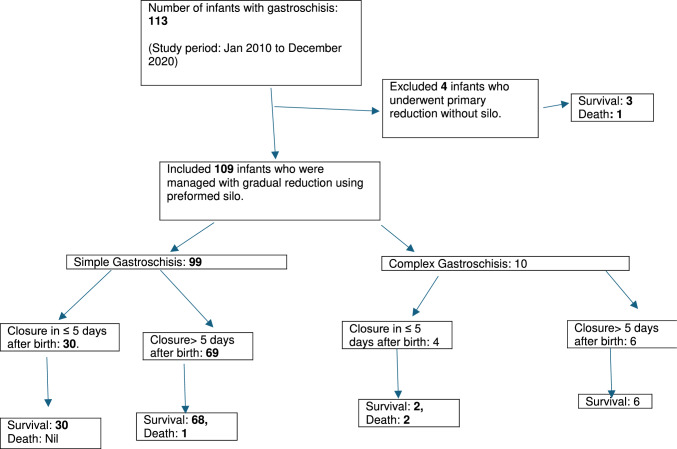
Flow diagram of study infants

In total, 113 live born infants with gastroschisis (January 2010–December 2020) were admitted to the unit during the study period. Four infants who underwent primary reduction without silo were excluded. The remaining 109 infants who were managed with the application of preformed siloes were included (Fig. 1). The median (IQR) gestation was 36.2 (35.2, 37) weeks and birth weight 2560 (2175, 2760) grams. All except one infant were diagnosed antenatally and only three were delivered in hospitals other than King Edward Memorial Hospital for Women, the only tertiary care perinatal centre in Western Australia (KEMH). All 113 infants were transferred to the neonatal intensive care unit of Perth Children's Hospital, immediately after birth. A total of 10 infants had complex gastroschisis. Of the 109 infants, 34 underwent early closure of abdominal defect and 75 underwent closure after five days. The median age at abdominal wall closure was 4 days (IQR: 3, 5) in the early group vs 7 days (IQR: 6, 10) in the late group (*p* < 0.0001). More number of infants in the late closure group had required large size silos and mesh patch to close the abdominal wall defect and needed pre-operative ventilation. Other baseline characters such as gestational age, birth weight and centiles, mode of delivery, antenatal diagnosis, age at the application of silo were similar between the two groups (Table 1).

**Table 1:** Clinical characteristics of neonates with gastroschisis managed with silo

	Total number of infants with gastroschisis *N* = 109	Abdominal wall closure within five days *N* = 34	Abdominal wall closure more than five days *N* = 75	*P*-value
Gestation at birth (weeks)	36.2 (35.2, 37)	36.3(35.4,37.1)	36.2(35,37)	0.586
Female	47/109 (43%)	15/34 (44%)	32/75 (43%)	1.000
Antenatal diagnosis	108/109 (99%)	33/34 (97%)	75/75 (100%)	0.312
Delivered at KEMH	106/109 (97.3%)	34/34 (100%)	72/75 (96%)	0.551
Mode of delivery	SVD: 49 (45%) Vacuum: 5 (4.6%) Forceps: 8 (7.3%) Elective LSCS: 7 (6.4%) Emergency LSCS: 40 (36.7%)	SVD: 18/34 (53%) Vacuum: 1/34 (2.9%) Forceps: 4/34 (11.8%) Elective LSCS: 2/34 (5.9%) Emergency LSCS: 9/34 (26.5%)	SVD: 31/75 (41.3%) Vacuum: 4/75 (5.3%) Forceps: 4/75 (5.3%) Elective LSCS: 5/75 (6.7%) Emergency LSCS: 31/75 (41.3%)	0.448
Birth weight (grams)	2560 (2175, 2760)	2645(2300,2770)	2540(2064, 2735)	0.138
Birth weight percentile	34 (16, 54)	33(19,54)	34(15,55)	0.673
Birth weight z score	− 0.40 (SD 0.95)	− 0.20(1.16)	− 0.47(0.83)	0.202
Birth length (cm)	47 (44, 48)	47(44,49)	46(43, 48)	0.197
Birth length centile	41 (20, 69)	45(25,75)	41 (17,64)	0.241
Birth length z score	− 0.19 (SD 1.20)	0.06(1.22)	− 0.31(1.19)	0.143
Birth head circumference (cm)	32 (31, 33.5)	32.5(30.5, 33.5)	32(31,33.5)	0.673
Birth head circumference centiles	40 (17, 63)	38.5 (24, 65)	41(17,62)	0.756
Birth head circumference z scores	− 0.16 (1.08)	− 0.28(0.94)	− 0.11(1.14)	0.412
Simple gastroschisis	99/109 (91%)	30/34(88%)	69/75 (92%)	0.499
Complex gastroschisis	10/109 (9%)	4(11.8%)	6(8%)	0.499
Known chromosomal anomalies	0/109	0/34 (0%)	0/75 (0%)	NA

*p* value of less than 0.05 was considered as statistically significant

*SD* Standard deviation, *IQR* Interquartile range, *SVD* Spontaneous vaginal delivery, *LSCS* Lower segment caesarean section, *KEMH* King Edward Memorial Hospital

### Comparison of outcomes between the early closure versus delayed closure group

The delayed closure group took longer time to commence enteral feeds [11 days (IQR: 9, 14) vs 6 days (IQR: 5, 8)], and longer time to reach full enteral feeds ([24.5 days (IQR: 17.5, 30) vs 15 days (IQR: 12, 22). They also had a longer duration of parenteral nutrition [24 days (IQR: 17, 29) vs 14 days (IQR: 20.5, 23.5)], longer duration of hospital stays [(32 days (IQR 23, 43) vs 19 days (IQR 15, 30) and a greater number of cumulative days on antibiotics [18 days (IQR 13, 24) vs 10 days (IQR: 8, 14)] (Table 2).

**Table 2:** Clinical management and outcomes during initial hospital stay

	All infants with gastroschisis *N* = 109 [A]	Abdominal wall closure within five days *N* = 34 [B]	Abdominal wall closure more than five days *N* = 75 [C]	*P*-value [B vs C]
Silo at KEMH	35/102 (34.3%)	14/33 (42.4%)	21/69 (30.4%)	0.269
Birth to silo application(hours)	2.71 (2.06, 3.6) *N* = 101	3.0(2.0, 3.7) *N* = 32	2.55(2.,3.6) *N* = 69	0.731
Silo size 3 4 5 6	3(3.2%) 23 (24.7%) 51 (54.8%) 16 (17.2%) *N* = 93	2(7.4%) 12 (44.4%) 13 (48.1%) 0 (0%) *N* = 27	1(1.5%) 11 (16.7%) 38 (57.6%) 16 (26.2%) *N* = 66	**0.001**
Age at abdominal wall closure (days)	6 (5, 8)	4 (3,5)	7 (6,10)	** < 0.0001**
Use of Mesh for closing the abdomen	28/108 (26%)	2/34 (5.9%)	26/74 (35.1%)	**0.001**
Preoperative ventilation	71/108 (65.7%)	18/34 (52.9%)	53/74 (71.6%)	0.080
Duration of ventilation (days)	3 (2, 6.5)	3 (1, 5)	4 (2,8)	**0.005**
Duration of ventilation in infants who were not ventilated pre-op (days)	2 (1, 3) *N* = 37	1.5 (1,3) *N* = 16	2 (2, 4) *N* = 21)	0.163
Duration of ventilation in infants who were ventilated pre-op (days)	5 (3, 8) *N* = 71	3.5 (2,5) *N* = 18	6 (3,9) *N* = 53	**0.044**
Number of days on antibiotics	15 (11, 22)	10 (8,14)	18(13,24)	** < 0.0001**
Age at commencing PN	1 (1, 1)	1 (1,1)	1(1,1)	0.909
Duration of PN (days)	21 (15, 29)	14 (10.5,23.5)	24(17, 29)	** < 0.0001**
Postnatal age at commencing enteral feeds	10 (7, 13)	6 (5,8)	11 (9,14)	** < 0.0001**
Post-op day at commencing enteral feeds after abdominal wall closure	3 (2, 4)	3 (2,3) *N* = 33	3 (3,4) *N* = 69	**0.004**
Postnatal age at full feeds (days)	21.5 (15.5, 29)	15 (12.5,22.5)	24.5 (17.5,30)	** < 0.0001**
Days taken to reach full feeds, once commenced	13 (10, 21)	11 (9, 19)	15 (11, 22)	0.091
Corrected gestational age at discharge (weeks)	40.4 (38.8, 41.2)	39.4(38, 40.5)	40.7(39.8, 41.4)	**0.0005**
Discharge weight (g)	3010 (2640, 3315)	2937(2460, 3150)	3010(2705, 3410)	0.121
Discharge weight centiles	7.5 (2.5, 25.5)	7.5(2.5,27.5)	7.5 (2.5,19.5)	0.821
Discharge weight z scores	− 1.36 (1.07)	− 1.24(1.24)	− 1.41(0.99)	0.510
Duration of hospital stay (days)	29 (20, 38.5)	19 (15, 30)	32 (23,43)	** < 0.0001**
Blood culture positive sepsis	16/108 (14.8%)	4/34(11.8%)	12/74(16.2%)	0.771
Abdominal compartment syndrome	3/108(2.8%)	0/34	3/74 (4%)	0.550
NEC	2/108 (1.8%)	1/34 (2.9%)	1/74 (1.3%)	0.533
Return to theatre during initial hospital stay	10/108 (9.2%)	2/34 (5.9%)	8/74 (10.8%)	0.500
Age at return to theatre (days)	44 (13, 61) range 1, 67 *N* = 11	26.5 (9, 44) *N* = 2	45(19, 61) *N* = 9	0.436
Mortality before discharge	3/109 (2.7%)	2/34 (5.9%)	1/75 (1.3%)	0.229

*p* value of less than 0.05 was considered as statistically significant

*PN* Parenteral nutrition, *NEC* Necrotising enterocolitis

### Clinical factors associated with age at reaching full enteral feeds

On univariable analysis, lower gestational age, lower birth weight, complex gastroschisis, the use of mesh patch, and delayed closure were associated with delayed age at reaching full enteral feeds. However, on multivariable analysis, only complex gastroschisis [ERC 2.30, CI:1.11, 3.73, *p* = 0.021] and delayed closure (ERC:1.40; CI:1.05, 1.86, *P* = 0.020] were associated with delayed age at reaching full enteral feeds (Table 3).

**Table 3:** Clinical factors associated with age at full feeds

	Univariable analysis		Multivariable analysis	
	Exponentiated Coefficient with 95% CI	P value	Exponentiated coefficient with 95% CI	*P* value
Gestational age	0.88 [0.81, 0.96]	**0.003**	1.60 [0.55, 4.67]	0.389
Birth weight	0.9997 [0.9994, 0.9999]	**0.010**	0.997 [0.993, 1.002]	0.317
Birth weight z score	0.96 [0.82, 1.12]	0.580	2.77 [0.35, 21.77]	0.332
Female sex	1.01 [0.77, 1.33]	0.941	0.89 [0.49, 1.61]	0.705
Complex gastroschisis	2.13 [1.25, 3.65]	**0.006**	2.03 [1.1.11, 3.73]	**0.021**
Use of mesh patch to close abdominal defect	1.42 [1.05, 1.93]	**0.022**	1.17[0.86, 1.60]	0.314
Preoperative ventilation	1.24[0.94,1.64]	0.127	1.03 [0.79, 1.35]	0.797
Days taken to commence feeds after abdominal wall closure	1.07 [0.99,1.15}	**0.097**	1.03 [0.96, 1.11]	0.389
Bowel in silo for more than five days prior to closing	1.49 [1.13,1.97]	**0.005**	1.40 [1.05, 1.86]	**0.020**

*p* value of less than 0.05 was considered as statistically significant

*CI* Confidence interval

### Clinical factors associated with duration of hospital stay

On univariable analysis, lower gestational age, lower birth weight, complex gastroschisis, the use of mesh patch, use of preoperative ventilation, and delayed closure were associated with prolonged hospital stay. However, on multivariable analysis, only complex gastroschisis (ERC: 1.69; CI: 1.10, 2.60, *p* = 0.017) and delayed closure (ERC: 1.41, CI: 1.11, 1.78. *P* = 0.005) were associated with prolonged hospital stay (Table 4).

**Table 4:** Clinical factors associated with length of hospital stay

	Univariable analysis		Multivariable analysis	
	Exponentiated Coefficient with 95% CI	*P* value	Exponentiated coefficient with 95% CI	*P* value
Gestational age	0.87 [0.81, 0.93]	** < 0.0001**	1.41 [0.59,3.36]	0.433
Birth weight	0.9995 [0.9993, 0.9997]	** < 0.0001**	0.998 [0.994, 1.001]	0.298
Birth weight z score	0.94 [0.82, 1.09]	0.424	2.26 [0.43, 11.94]	0.335
Female sex	1.09 [0.85, 1.39]	0.508	0.88 [0.55, 1.43]	0.613
Complex gastroschisis	1.78 [1.22, 2.61]	**0.003**	1.69 [1.10, 2.60]	**0.017**
Use of mesh patch to close abdominal defect	1.34 [1.02, 1.75]	**0.033**	1.09 [0.85, 1.40]	0.491
Preoperative ventilation	1.29 [1.01,1.66]	**0.043**	1.05 [0.84, 1.30]	0.694
Days taken to commence feeds after abdominal wall closure	1.07 [0.99, 1.14]	**0.058**	1.02 [0.97, 1.08]	0.395
More than five days in silo prior to closing	1.64 [1.29, 2.10]	** < 0.0001**	1.41 [1.11, 1.785]	**0.005**

*p* value of less than 0.05 was considered as statistically significant

*CI* Confidence interval

### Mortality

Three infants died during initial hospital stay (two infants in the early closure and one in the delayed closure group). The first infant was born at 34.5 weeks gestation and weighed 2120 g. She had complex gastroschisis with narrow mesentery and volvulus and necrotic bowel that was noticed immediately at birth. She was provided palliative care and passed away on postnatal day four. The second infant was born at 31 weeks' gestation and weighted 1690 g. He underwent silo application within 2 h of birth. Silo closure was done on day 39 and required the use of mesh to close the abdomen. He had prolonged feed intolerance, pulmonary hypertension, and recurrent sepsis. He needed 45 days of antibiotics (six courses in total) and succumbed to multiple complications on postnatal day 69. The third infant was born at 35 weeks' gestation and weighed 2560 g. He had complex gastroschisis, with necrotic small bowel noticed immediately at birth. The gut was considered not salvageable and hence the infant was palliated and died on postnatal day 4.

### Gut necrosis

Two infants developed gut necrosis during initial hospital stay; the first infant was born at 36 weeks' gestation and weighed 2300 g at birth. She had simple gastroschisis and underwent silo placement within few hours of birth and surgery on day 5. She had 48 days of antibiotics (6 courses), and 54 days of TPN. She developed wound infection, central line infection and had prolonged feed intolerance. She underwent laparotomy and resection of stenotic ileal segment, which was thought to be post- subacute NEC, and made good recovery. The second infant was born at 35-week gestation and weighed 2900 g. He had simple gastroschisis and underwent silo placement within few hours after birth. Abdominal wall closure was done on day 7, but developed NEC, and klebsiella sepsis, candida wound infection, and bowel obstruction secondary to adhesions at 2 months of age. In total, he received 60 days antibiotics, 54 days TPN and attained full enteral feeds at 70 days of postnatal age.

## Discussion

Our retrospective study reports on the outcomes of neonates with gastroschisis managed using staged reduction with preformed silos in our unit. The mortality rate was very low (3/109; 2.7%) and compared favourably with other recent publications [11–14] that have reported mortality rates of 1 to 10%. In two of the infants who died in our cohort, the gut was extensively necrotic at birth and hence were offered palliative care. The third infant was very preterm (31w) and succumbed to pulmonary hypertension and multiple infections after prolonged hospital stay.

The median 29 (IQR 20, 38.5) days of hospital stay is similar to or better than the published literature [15–21]. Most importantly, none of the infants managed with silo reduction developed features of acute abdominal compartment syndrome.

Currently there is limited evidence from RCTs as to whether staged reduction using silo or primary reduction is preferable in neonates with gastroschisis. One systematic review found beneficial effects of preformed silos [22] whereas another one concluded that there was no strong evidence to support a preference for any strategy [23]. The American Pediatric Surgical Association has recommended that primary repair (i.e. without silo should be attempted when physiological status and abdominal domain permit [24]. Only one study in their review was a RCT (*n* = 38) [14] and it had not included the other RCT(*n* = 27) [6]. A recent retrospective multicentre cohort study by the Children’s Hospitals National Consortium (CHNC) in the USA reported that nearly 65% of infants were managed with staged reduction [25].

Whilst staged reduction with preformed silos is an excellent way of managing gastroschisis, there is a possibility that if the final closure of abdominal wall defect is delayed too long, it can result in increased morbidities. We found that, delaying the abdominal wall closure beyond five days after birth was associated with prolonged time to reach full enteral feeds and prolonged hospital stay. These results remained significant even when potential confounders such as gestation, complex gastroschisis, and size of abdominal wall defect were adjusted for.

Our results concur with those of Hawkins et al. [9] who found that silo closure beyond five days was associated with prolonged hospital stay, delayed time to reach full enteral feeds and the duration of ventilation and TPN. We concur with their proposal that closure within 5 days can avoid many of the risks commonly attributed to delay in closure [9]. Hence, every attempt should be made to complete the silo reduction and close the abdominal wall defect within five days after birth.

The strengths of our study are the large sample size, and the inclusion of all infants (simple gastroschisis, complex gastroschisis, term gestation, preterm gestation) and the use of standardised protocol for management. The other strength is the use of multivariable regression analyses to adjust for important confounders. To our knowledge, it is the first study that has compared the outcomes of early versus delayed closure of the abdominal wall in neonates with gastroschisis managed with preformed silos. The main weakness of our study is the retrospective nature and inability to adjust for unknown, but important confounders. Hence, the results should be interpreted cautiously. Moreover, since the results are from a single centre from a high-income country, further research is needed in other settings. Future studies should be of prospective design, preferably adequately powered RCTs.

## Conclusions

Gradual reduction of herniated viscera using preformed silo results in good outcomes with minimal risk of abdominal compartment syndrome in neonates with gastroschisis. However, delaying the closure of abdominal wall beyond five days was associated with longer duration of hospital stay and longer time taken to achieve full enteral feeds. Completing the silo reduction and closing the abdominal wall defect within five days may further improve their outcomes.

## Data Availability

Data will be made available by the correspondence author on reasonable request.
